# Risk factors for developing anorectal dysfunction after anterior resection

**DOI:** 10.1007/s00384-021-04024-3

**Published:** 2021-09-02

**Authors:** Kevin Afshari, Kenneth Smedh, Philippe Wagner, Abbas Chabok, Maziar Nikberg

**Affiliations:** 1 Colorectal Unit, Department of Surgery and Centre for Clinical Research of Uppsala University, Västmanland’s Hospital Västerås, 72189 Västerås, Sweden; 2 Centre for Clinical Research, Uppsala University, Hospital of Vastmanland Västerås, Västerås, Sweden

**Keywords:** Anorectal dysfunction, Anterior resection syndrome, Functional bowel disturbance, Anterior resection, Bowel disturbance, Functional outcome, Bowel dysfunction

## Abstract

**Background:**

Anterior resection (AR) may result in defecatory dysfunction and the cause is multifactorial. The aim was to explore if dysfunction could be related to the part of the colon used for anastomosis (sigmoid or descending) and to identify other possible risk factors for bowel dysfunction after AR.

**Methods:**

This is a retrospective study based on prospectively registered data from a regional registry at the surgical department in Västmanland 1996–2019. Bowel function was registered at 1 year after AR or after stoma reversal. In total, 470 stage I–III rectal cancer patients had AR whereof 412 were included in this study.

**Results:**

Clustering was seen in 57%, incontinence 29%, urgency 22%, and evacuatory dysfunction 16%. The part of the colon used for anastomosis, level of vascular tie, and gender were not significantly associated with defecatory dysfunction. The higher the anastomotic level, the lower the risk of incontinence (OR 0.75; CI 0.63–0.90; *p* < 0.001) and clustering (OR 0.78; CI 0.67–0.90; *p* < 0.001). Compared with patients without a loop-ileostomy, an increased risk of clustering (OR 1.89; 1.08–3.31; *p* = 0.03), incontinence (OR 2.48; 1.29–4.77; *p* < 0.01), and urgency (OR 4.61; CI 2.02–10.60; *p* < 0.001) was seen after loop-ileostomy closure. Preoperative radiotherapy had a negative impact on continence and clustering seen mainly in the unadjusted analysis.

**Conclusion:**

The part of the colon used for anastomosis was not a significantly associated functional outcome after anterior resection. Low anastomotic level and having had a diverting ileostomy were independent risk factors associated with negative functional outcomes.

**Supplementary Information:**

The online version contains supplementary material available at 10.1007/s00384-021-04024-3.

## Introduction

The gold standard for the treatment of adenocarcinoma in the rectum has been the sphincter-sparing anterior resection (AR) [1]. However, there are long-term side effects of which defecatory dysfunction including incontinence for feces and flatus, urgency, diarrhea, frequency, and clustering are the most common. This combination of symptoms is recognized as low anterior resection syndrome (LARS) [2]. Major LARS is reported in 18 to 56% and the symptoms appear directly after surgery or after closure of a diverting stoma [3]. Symptoms may improve with time but about 12 to 18 months postoperatively the symptoms reach a plateau and further improvements are unlikely [4, 5]. In a recent multicenter study, exploring quality of life in patients with LARS, it was determined that quality of life was impaired in patients with major LARS even up to 16 years after surgery [6].

The cause of LARS is multifactorial and may be due to damage to the anal sphincter [7, 8], neural damage during pelvic dissection [7, 8], and altered colonic and neorectal motility [9]. Other identified risk factors for developing LARS are anastomotic height, anastomotic type, total mesorectal excision (TME) versus partial mesorectal excision (PME), adjuvant and neoadjuvant radiotherapy, complications such as anastomotic leakage, and diverting ileostomy [1, 3, 10–13].

Yet another factor which could be associated with LARS is the level of transection of the colon that is anastomosed to the anorectal stump. The sigmoid colon could be more rigid with a narrower lumen and with more diverticula compared to the descending colon, which might result in different functional outcomes. To our knowledge, it is not known if the functional outcome is affected whether the sigmoid or descending colon is anastomosed to the anorectum when performing low anterior resection. The primary aim was to explore if rectal cancer patients operated with AR will have poorer bowel function when the sigmoid colon is used for anastomoses compared with the descending colon. Secondarily, we aimed to identify other possible risk factors for bowel dysfunction after AR.

## Methods

This study was based on retrospective analysis of prospectively collected data from a regional population-based registry on all operated patients with rectal cancer diagnosed between January 1996 and January 2019 in the Västmanland County. The data set includes details on pre-, peri-, and postoperative data. Patients were followed up with clinical examinations at 1, 6, 12, 24, 36, 48, and 60 months after surgery and the bowel function was registered prospectively (defecatory frequency, urgency, incontinence for flatus and stool, pad usage, clustering/fragmentation, and evacuatory dysfunction) by a colorectal surgeon or a trained nurse specialist using a questionnaire (see Supplementary) [14]. The data from the questionnaire was then registered in the database by a research nurse. The questionnaire of bowel function was set up in 1996 and before the development of the LARS score and did not include any quality of life questions.

A rectal adenocarcinoma was defined as a tumor with its distal margin within 15 cm from the anal verge measured with a rigid rectoscope. Preoperative screening for metastases was routinely performed. Up until 2002, chest radiography and liver ultrasonography were used, and thereafter, computed tomography of the thorax and abdomen. Magnetic resonance imaging of the rectum was used routinely from 1996 [15]. Stage was defined according to the 6th American Joint Committee on Cancer (AJCC) TNM classification.

### Study population

**Fig. 1:**
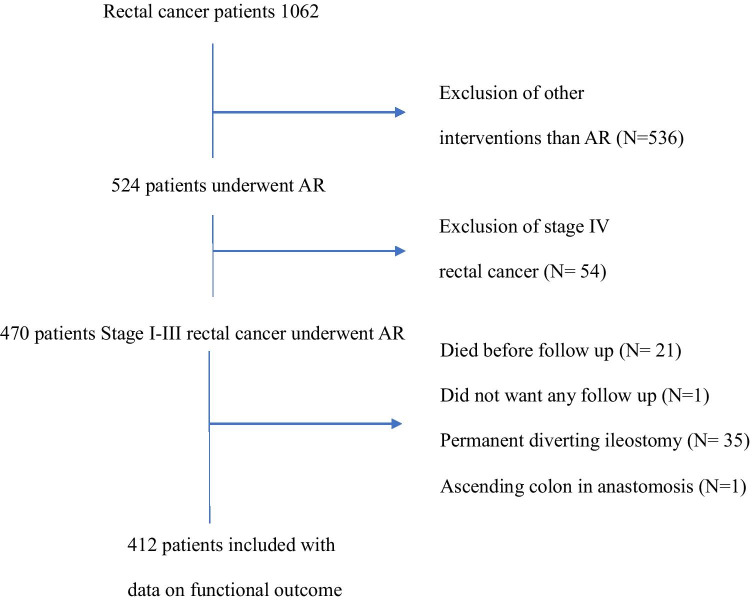
Flowchart of the selection process of rectal cancer patients undergoing anterior resection in the county of Vastmanland, Sweden, 1996–2019

During the study period, 1062 patients were diagnosed with rectal cancer, whereof 524 underwent AR. In total, 470 patients were stage I–III, after exclusion of primary metastasized rectal cancer (*N* = 54). Data on bowel function were available in 412 patients as 21 patients died before follow-up, one refused follow-up, one had an anastomosis between the ascending colon and rectum, and in 35 patients the diverting stoma became permanent (Fig. 1).

In Table 1, data on the whole cohort with stage I–III rectal cancer who underwent AR is presented (*N* = 470). Tables 2, 3, and 4 present data on functional outcomes in the 412 patients that constituted the study group.

**Table 1:** Demographics, patient, and surgical characteristics of stage I–III rectal cancer patients undergoing anterior resection for rectal cancer (*N* = 470) after exclusion of primary metastasized rectal cancer

Variables	Total patients *N* = 470
**Age (year)**^**#**^	68 (32–86)
**Gender (male:female) (%)**	279:191 (60:40)
**BMI (kg/m** [2]**)**^**#**^	26 (16–43)
**ASA** 1–2 3 4	365 (78) 105 (22) 0 (0)
**Tumor distance from the anal verge** Low (3–5 cm) Middle (6–10 cm) High (11–15 cm)	51 (11) 230 (49) 189 (40)
Radiotherapy* Yes No	320 (68) 150 (32)
**Minimal invasive surgery** Yes No	37 (8) 433 (92)
**Resection of other organs** Yes No	75 (16) 395 (84)
**Central ligature IMA** Yes No Missing	419 (89) 49 (10) 2 (1)
**Mobilization of the splenic flexure** Yes No	407 (87) 63 (13)
**Type of anastomosis** Colonic reservoir End-to-side colo-anal End-to-side colo-rectal	77 (16) 330 (70) 63 (14)
**Part of the colon used for anastomosis** Sigmoid Descending Transverse Ascending	139 (29) 319 (68) 11 (2) 1 (1)
**Anastomotic level (cm)**^**#**^	4.5 (2–11)
**Type of surgery** TME PME	422 (90) 48 (10)
**Diverting ileostomy** Yes No	352 (75) 118 (25)
**T-stage** 0–2 3–4	200 (43) 270 (57)
**N-stage** 0–x 1–2	293 (62) 177 (38)
**Anastomotic leakage** Yes No	33 (7) 437 (93)
**Re-laparotomy** Yes No	15 (3) 454 (97)
**Adjuvant chemotherapy** Yes No Missing	186 (40) 269 (57) 15 (3)

*ASA*, American Society of Anesthesiologists; *BMI*, body mass index; *IMA*, inferior mesenteric artery; *TME*, total mesorectal excision; *PME*, partial mesorectal excision

*Continuous values are presented as median (range)

Other values in number of patients and parentheses are percentages

**Radiotherapy is any radiotherapy given at any time prior to surgery for rectal cancer, including radiotherapy for cancers other than rectal cancer. Four patients had radiation due to other previous cancer

**Table 2:** Major complications after anterior resection and complications leading to re-laparotomy

**Complications**	*N* = 43	Re-laparotomy (*N* = 15)
Anastomotic leakage	33 (7)	13
Small bowel obstruction	4 (1)	1
Stomal complication	5 (1)	1
Urinary bladder tamponade	1 (0.2)	

Values in parentheses are percentages

**Table 3:** Overview of functional outcome in 412 patients, stage I–III, after anterior resection

**Incontinence** Yes No Missing	123 (29) 283 (69) 6 (2)
**Evacuatory dysfunction** Yes No Missing	65 (16) 336 (81) 11 (3)
**Clustering** Yes No Missing	234 (57) 154 (37) 24 (6)
**Urgency** Yes No Missing	91 (22) 310 (75) 11 (3)

Values in number of patients and parentheses are percentages

**Table 4:** Univariable logistic regression analysis of functional outcome and risk factors in patients undergoing anterior resection for rectal cancer in Västmanland County (*N* = 412)

	**Incontinence** **(*N* = 406)**			**Urgency** **(*N* = 401)**			**Evacuatory dysfunction** **(*N* = 401)**			**Clustering** **(*N* = 388)**		
	**OR**	**95% CI**	***P***	**OR**	**95% CI**	***P***	**OR**	**95% CI**	***P***	**OR**	**95% CI**	***P***
**Age**	**1.03**	**1.00**–**1.05**	**0.02**	0.99	0.96–1.01	0.31	0.99	0.96–1.02	0.50	**0.97**	**0.95**–**0.99**	**0.01**
**Gender** Kvinna Man	1 1.12	0.73–1.73	0.60	1 1.26	0.78–2.05	0.35	1 0.92	0.54–1.58	0.77	1 0.93	0.61–1.40	0.71
**BMI**	0.96	0.91–1.01	0.13	**1.06**	**1.00**–**1.12**	**0.048**	0.98	0.92–1.05	0.65	1.03	0.98–1.08	0.30
Radiotherapy* No Yes	**1** **1.69**	**1.03**–**2.69**	**0.04**	1 1.61	0.94–2.74	0.08	1 1.85	0.98–3.48	0.06	**1** **2.10**	**1.36**–**3.25**	** < 0.001**
**Central ligature IMA** No Yes	1 1.25	0.61–2.60	0.55	1 2.28	0.87–5.98	0.10	1 1.90	0.65–5.53	0.24	1 1.30	0.68–2.45	0.43
**Anastomotic level**	**0.71**	**0.60**–**0.83**	** < 0.001**	0.92	0.80–1.06	0.24	0.86	0.72–1.02	0.08	**0.75**	**0.66**–**0.85**	** < 0.001**
**Part of the colon used for anastomosis** Descending Sigmoid	1 0.99	0.62–1.56	0.95	1 0.58	0.34–1.01	0.05	1 0.71	0.39–1.30	0.27	1 1.17	0.75–1.81	0.50
**Diverting ileostomy** No Yes	**1** **3.12**	**1.74**–**5.58**	** < 0.001**	**1** **4.09**	**1.97**–**8.47**	** < 0.001**	1 1.69	0.86–3.30	0.13	**1** **2.62**	**1.63**–**4.20**	** < 0.001**
**Adjuvant chemotherapy** No Yes	**1** **0.61**	**0.39**–**0.95**	**0.03**	1 1.31	0.82–2.10	0.26	1 0.81	0.47–1.40	0.44	**1** **1.59**	**1.04**–**2.42**	**0.03**
**Anastomotic leakage** No Yes	1 2.40	0.93–6.21	0.07	1 2.50	0.92–6.77	0.07	1 2.07	0.71–6.02	0.18	1 2.21	0.71–6.90	0.17

*CI*, confidence interval; *OR*, odds ratio

### Surgery

Anterior resection was standardized with either partial mesorectal excision (PME) in tumors in the upper rectum or total mesorectal excision (TME) in middle or low rectal tumors and only performed in the hands of a few colorectal surgeons. Tumors located less than 3–4 cm from the anal verge did not have an anastomosis due to our local policy, in fear of poor functional results. Central ligation of the inferior mesenteric artery (IMA) 1–2 cm from the aorta or ligation at the superior rectal artery (SRA) close to the origin of the left colic artery (LCA) was performed routinely [16]. Sigmoid, descending, or transverse colon was used for an anastomosis. Which part used depended on the length of the colon, the arterial circulation, the quality of the bowel wall, and the presence of multiple diverticula. The part of the colon having an S shape is defined as the sigmoid colon and the straight part of the left colon was defined as the descending colon*.* Between the years 1996 and 2002, only a colonic pouch was used for anastomosis, and thereafter only an end-to-side anastomosis was performed as the standard anastomotic type after LAR. For PME, only end-to-side colorectal anastomosis was used. No straight anastomosis was constructed. The part of the colon used and all other perioperative data were prospectively registered by the surgeons at the time of surgery. Laparoscopic rectal cancer surgery was introduced in the year 2014 and robotic surgery in the year 2016.

### Data variables

Patients were preoperatively asked about defecation habits before symptoms and diagnosis of rectal cancer. Functional outcomes after rectal cancer surgery were registered as dichotomous variables at each follow-up. Incontinence was defined as leakage more than once a week, urgency as a sudden need of defecation, evacuatory dysfunction as defecation lasting more than 15 min with or without an enema, and clustering/fragmentation as the need of going to the lavatory within 30 min after defecation (see Supplementary). The functional data after a minimum of 12 months after primary surgery for rectal cancer was used, as evidence has shown that bowel dysfunction becomes more stable 12 months after anterior resection [17, 18]. For patients who received a diverting loop-ileostomy at the primary surgery or within the first 30 days postoperatively due to complications, functional data was registered 12 months after closure of the diverting stoma. Evacuatory dysfunction was added to the follow-up form at a later stage when some patients had missing values.

To avoid sparse variable strata, the ASA scores were recoded into dichotomous variables (ASA 1–2 versus ASA 3–4). The T-stage (T0-2 versus T3 versus T4) and N-stage (N0 versus N1–N2) scores were combined in a similar manner.

Both laparoscopic surgery and robotic surgery were registered as minimal invasive surgery.

In the analysis of the type of colon anastomosed, the remaining part of the descending and the left part of the transverse colon used in anastomosis were merged as one group.

Anastomotic leakage was defined as any clinical signs of leakage, confirmed by radiological examination or endoscopic and clinical examination of the anastomosis. Colo-vaginal fistula and pelvic abscess were registered as anastomotic leakage.

### Statistical analysis

Continuous data were reported as mean ± standard deviation (SD) or median with range. Categorical data were analyzed for differences in proportions using the *χ* [2] test or Fisher’s exact test for low numbers. Univariable and multivariable analyses for factors affecting functional data were performed using logistic regression with goodness of fit evaluated using the Hosmer–Lemeshow test. Collinearity of independent variables in the logistic regression was investigated using the variance inflation factor. Data were analyzed using SPSS software (v. 26; IBM Corp., Armonk, NY, USA).

## Ethics

The study was approved by the Regional Ethics Review Board in Uppsala and complied with the Declaration of Helsinki (Dnr 2014/389 and 2020–05,140).

## Results

The characteristics of all 470 patients with stage I–III who underwent anterior resection with PME or TME for rectal cancer are summarized in Table 1. The mean age was 67 ± 9 years. Sixty percent were male, and the majority were ASA 1–2. Sixty-eight percent (*n* = 320) received preoperative radiotherapy (RT) of which four had previous radiation due to prostatic or gynecological cancer prior to the rectal cancer diagnosis. Short preoperative RT (5 × 5 Gy) was given to 57% (*N* = 268) and 10% (*N* = 48) had long preoperative RT (1.8–2 × 25 Gy). Most anterior resections were performed with open surgery, of which 90% (*n* = 422) constituted TME. The descending colon was used in 68% (*N* = 319) when creating the anastomosis and an end-to-side colo-anal anastomosis was performed in 70% (*N* = 330). Seventy-five percent (*N* = 352) received a diverting ileostomy. Postoperative major surgical complications within 30 days were seen in 9% (*N* = 43), of which 35% (*N* = 15) underwent re-laparotomy due to the complications (Table 2). Mortality within 30 days was 0.4% (*N* = 2) and 90-day mortality was 1% (*N* = 6).

### Functional outcome

When patients preoperatively were asked about defecation habits before symptoms and diagnosis of rectal cancer, 68% of patients (*N* = 282) reported “normal” defecation (1–2 per day), 4% defecated every other day (*N* = 16), 4% (*N* = 16) had varying stool consistency, and 8% (*N* = 31) had loose stool more than 3 per day.

Minimum a year after primary surgery or closure of the diverting ileostomy, the median stool frequency was 3 per day (range 0–11). Perianal skin irritation was observed in 10% (*N* = 42). Due to change in bowel habits, 10% (*N* = 40) used motility-promoting medications, 29% (*N* = 121) used inhibitory medications, and 7% (*N* = 27) used a combination of these. Pad usage was registered in 39% (*N* = 162). The most common functional defecatory outcome is clustering (57%) and incontinence (29%) followed by urgency (22%) and evacuatory dysfunction (16%) (Table 3).

### Risk factors for functional outcome

With age, the risk of incontinence increased by 3% and equally reduced for clustering in the univariable analysis (Table 4), but in the multivariable analysis, age was only significantly associated with clustering (Table 5). Gender showed limited association with functional difficulties in the current study. The anastomotic level had significant association with defecatory outcome. The higher anastomoses, in relation to the anal verge, the lower the risk in both the univariable and multivariable analyses of both incontinence (OR 0.75; CI 0.63–0.90; *p* < 0.001) and clustering (OR 0.78; CI 0.67–0.90; *p* < 0.001) (Tables 4 and 5). The part of the colon, sigmoid or descendening, that was used for anastomosis was not significantly associated with any functional outcome (Tables 4 and 5). Neither was there an association seen on defecatory function regarding level of vascular ligature (Tables 4 and 5).

**Table 5:** Multivariabel logistic regression analysis of functional outcome and risk factors in patients undergoing anterior resection for rectal cancer in Västmanland County (N = 412)

	**Incontinence** **(*N* = 406)**			**Urgency** **(*N* = 401)**			**Evacuatory dysfunction** **(*N* = 401)**			**Clustering** **(*N* = 388)**		
	**OR**	**95% CI**	***P***	**OR**	**95% CI**	***P***	**OR**	**95% CI**	***P***	**OR**	**95% CI**	***P***
**Age**	1.02	1.00–1.05	0.09	0.99	0.96–1.02	0.38	0.99	0.96–1.02	0.38	**0.97**	**0.95**–**1.00**	**0.02**
**Gender** Kvinna Man	1 1.06	0.66–1.70	0.82	1 1.10	0.65–1.85	0.73	1 0.86	0.49–1.53	0.61	1 0.86	0.54–1.37	0.52
**BMI**	0.97	0.92–1.04	0.40	1.05	0.99–1.12	0.13	0.99	0.92–1.06	0.76	1.03	0.97–1.09	0.40
Radiotherapy* No Yes	1 1.16	0.67–2.00	0.60	1 1.14	0.62–2.08	0.68	1 1.52	0.77–3.03	0.23	1 1.45	0.87–2.40	0.15
**Central ligature IMA** No Yes	1 1.37	0.58–3.21	0.47	1 2.02	0.69–5.90	0.20	1 1.43	0.44–4.65	0.55	1 1.56	0.70–3.48	0.28
**Anastomotic level**	**0.75**	**0.63**–**0.90**	** < 0.001**	1.02	0.86–1.22	0.81	0.94	0.78–1.14	0.54	**0.78**	**0.67**–**0.90**	** < 0.001**
**Part of the colon used for anastomosis** Descending Sigmoid	1 1.18	0.68–2.05	0.57	1 0.67	0.36–1.25	0.21	1 0.78	0.39–1.56	0.49	1 1.71	0.98–2.98	0.06
**Diverting ileostomy** No Yes	**1** **2.48**	**1.29**–**4.77**	**0.01**	**1** **4.61**	**2.02**–**10.6**	** < 0.001**	1 1.45	0.69–3.03	0.33	**1** **1.89**	**1.08**–**3.31**	**0.03**
**Adjuvant chemotherapy** No Yes	1 0.74	0.46–1.20	0.22	1 1.20	0.72–2.01	0.49	1 0.77	0.43–1.37	0.37	1 1.51	0.94–2.40	0.09
**Anastomotic leakage** No Yes	1 1.75	0.66–4.66	0.26	1 2.07	0.74–5.81	0.17	1 1.75	0.59–5.24	0.32	1 1.93	0.59–6.29	0.28

*CI*, confidence interval; *OR*, odds ratio

In median, the time from stoma creation to closure was 8 months (range 1–29 months). After closure of the diverting ileostomy, there was a significant increased risk of clustering (OR 1.89; 1.08–3.31; *p* = 0.03), incontinence (OR 2.48; 1.29–4.77; *p* < 0.01), and urgency (OR 4.61; CI 2.02–10.60; *p* < 0.001) in the multivariable analysis (Tables 4 and 5). Anastomotic leakage was not associated with any functional difficulties in neither the univariable nor the multivariable analysis (Tables 4 and 5). Radiotherapy given prior to surgery and postoperative adjuvant chemotherapy was not significant in the multivariable analysis (Table 5).

Goodness-of-fit tests for all logistic regression models used for risk factor analyses were all non-significant, indicating satisfactory fit of all models.

## Discussion

Anterior resection for rectal cancer is closely linked with functional outcome in patients. The part of the colon used for anastomosis—descending or sigmoid colon—was not a significantly associated functional outcome after anterior resection in this study. However, low anastomotic level was associated with incontinence and clustering. Anastomotic leakage was not observed to covary with intestinal function but receiving a diverting ileostomy increased the risk of having functional difficulties 1 year after closure. Gender and pre- or postoperative oncological treatment were not associated with intestinal function but the risk for clustering decreased with increasing age.

The primary aim was to examine if sigmoid or descending colon used in anastomosis led to worse functional outcome. The sigmoid compared with the descending colon could be more rigid with a narrower lumen and may have multiple diverticula, which might hypothetically result in defecatory dysfunction. We did not find level of intestinal transection to be significantly associated with functional outcome. However, the sigmoid colon was only used for anastomoses when it was assessed during surgery to have a normal bowel thickness without multiple diverticula and the transection line was always governed by the arterial circulation at the distal bowel end.

When performing low anterior resection, the vascular ligation is either centrally at the IMA close to the aorta or peripheral at the superior rectal artery at varying levels. There is no consensus where the preferred vascular ligation should be regarding functional outcome postoperatively. Central ligation may cause sympathetic nerve damage causing fecal incontinence as well as urinary and sexual disturbances [19]. In a study on the Swedish national registry, no difference was found between central or peripheral ligature on the risk of defecatory disturbances 2 years after anterior resection [20]. There are two randomized trials with discrepant results: one which did not find any difference in risk for defecatory disturbances regardless of level of ligation [21] and the other which found increased stool frequency after central ligation [22]. We did not find an association between functional outcome and level of vascular ligation in this study, probably because at our department the mean difference between a central ligation and the peripheral ligation, which is done close to the origin of the left colic artery, was less than 1 cm [16].

Low anastomotic level increased the risk of incontinence and clustering. This is in accordance with some studies, where low anastomotic height was associated with major low anterior resection syndrome (LARS) [2, 18, 23–26]. It has been suggested that functional outcome after rectal resection is due to reduced neorectal compliance when constructing a low anastomosis [9, 24, 27]. Thus, probably, it is important to preserve as much rectum as possible to reduce risk of functional difficulties after surgery.

Today, it is standard practice to use a diverting ileostomy following low anterior resection, to reduce the consequences of an anastomotic leak [28]. However, fashioning a diverting ileostomy has been shown to have negative consequences on the bowel function in several studies [12, 27, 29, 30]. Our results show an increased risk of incontinence, urgency, and clustering after 12 months of diverting ileostomy closure. There are emerging studies indicating timing of stoma closure to be important for bowel function with cutoffs at approximately 3 months [18, 30, 31] or 6 months [1]. The median time to closure in this study was 8 months, maybe explaining the association to bowel dysfunction.

In a recent meta-analysis of LARS and risk factors, having anastomotic leakage was found, in several studies, to be associated with increased risk of major LARS (3). However, some studies have not shown increased risk following anastomotic leakage [1, 18, 24], in accordance with our findings. The possible discrepancy in the results is due to different definitions of anastomotic leakage and size of cohorts. In this cohort, a type II error may explain why anastomotic leakage was not associated with significant increase risk of incontinence in the multivariable analysis.

Preoperative RT has consistently been shown to be a risk factor for LARS in studies, both after short and especially long courses [1, 2, 18, 23–26]. In our study, the majority had short-course preoperative RT with a negative impact on continence and clustering in the univariable analysis, but not in the multivariable analysis when adjusted for anastomotic level. This is in accordance with a recent Scandinavian study where radiotherapy as a risk factor for LARS was abolished when adjusted for tumor height [32]. Another possible explanation for the lack of association between RT and bowel function could be due to the number of variables used in the multivariable analysis, reducing power in the analysis.

The main strengths of this study are that the data were registered prospectively, which limits the bias associated with retrospectively collected data. The data are based on a large population-based homogenous cohort and included all patients with rectal cancer operated with anterior resection, in the county since 1996. Furthermore, there could be benefits of a single-center study, such as surgery being standardized and performed by a few experienced colorectal surgeons, with the same approach to treatment of rectal cancer patients, consensus on the definitions of all variables, as well as all variables being registered at each follow-up visit in a protocol by the same nurse and surgeons.

The main limitation of this study concerning choice of colon segment for anastomosis could be its non-randomized design and the lack of exact distance of the transection level of the sigmoid or descending colon to the anal verge. However, as for practical reasons, a randomization would be difficult as several technical factors are involved such as status of bowel segment and arterial circulation. Furthermore, the data were not based on the validated LARS score not fully comparable to studies on LARS; however, the functional outcome variables selected cover almost the same symptoms as in the LARS score. The questions in the questionnaire were asked by the treating surgeons and nurse, maybe introducing response bias and affect the validity of the questions; however, the same method was used for all patients why the results should still be valid. Finally, for the variable evacuatory dysfunction, conclusions from this variable should be made cautiously due to low statistical precision and subsequent confidence interval width.

## Conclusion

The part of the colon used for anastomosis—descending or sigmoid colon—was not a significantly associated functional outcome after anterior resection when the quality of the bowel wall and adequate distal arterial blood circulation are taken into consideration in the present study, although further studies are needed to settle this question. Low anastomotic level is associated with incontinence and clustering and having had a diverting ileostomy is also associated with functional difficulties after stoma reversal. Patients should be given this information when confirming their consent for low anterior resection.

## Supplementary Information

Below is the link to the electronic supplementary material.

Supplementary file1 (DOCX 14 KB)
