# Supplementary material for: Risk factors for developing anorectal dysfunction after anterior resection
Source: Int J Colorectal Dis. 2021 Sep 2;36(12):2697–705. doi: 10.1007/s00384-021-04024-3 (PMC8589768; doi:10.1007/s00384-021-04024-3)
Supplement: Supplementary file 1 — Supplementary file1 (DOCX 14 KB) [file 384_2021_4024_MOESM1_ESM.docx]

**Supplementary – Questionary used at diagnosis and follow up regarding bowel function**

**Questionary at diagnosis regarding bowel function before the rectal cancer diagnosis**

*Do you have “normal” defecation (1-2 per day)?*

*Do you defecate every other day?*

*Do you have varying stool consistency (loose-firm)?*

*Do you have loose stool more than 3 per day?*

*For how long did you have the symptoms:*

*<1month*

*1-3months*

*3-6months*

*>6 months*

*No local symptoms*

**Questionary at follow up regarding bowel function outcome**

The variables beneath are registered at the following months after primary surgery:

6, 12, 24, 36, 48, 60

**Incontinence ( >1/week):**  *Do you have leakage more than once a week? Yes/No*

**Urgency:** *Do you ever fell a sudden need to defecate? Yes/No*

**Evacutory dysfunction (>15 minutes in the toilet and/or need to use enema):** *Do you sit more than 15 min in the bathroom to empty your bowel? Yes/No*

**Clustering (need to go to the toilet <30minutes after defecation):** *Do you need to go to the lavatory within 30 minutes after defecation to defecate again? Yes/No*

**Pad usage:** *Do you use pads regularly? Yes/No*

**Perineal skin irritation:** *Yes/No*

**Stool frequency:** *Number of stools/day*

**Medication for bowel movement regulation:** *Do you use medication to regulate your bowel movement? If yes what medication do you use*
